# Beyond the Virological Benefits of the Herpes Zoster Vaccine in the Context of Primary Care

**DOI:** 10.3390/vaccines14010079

**Published:** 2026-01-11

**Authors:** Carlo Fabris, Lorena De Cecco Beolchi, Lucia Casatta, Stefano Celotto, Marina Pellegrini, Serafina Lovascio, Katia Urli, Pierluigi Toniutto

**Affiliations:** 1 Hepatology and Liver Transplantation Unit, Azienda Sanitaria Universitaria Integrata, University of Udine, Faculty of Medicine, 33100 Udine, Italy; carlofabrisudine@libero.it; 2 Center of Formation in General Medicine (CEFORMED), 34074 Monfalcone, Italy; lorena.dececco@asfo.sanita.fvg.it; 3 Health District of Udine, Azienda Sanitaria Universitaria Integrata Friuli Centrale, 33100 Udine, Italy; lucia.casatta@karimsrl.it (L.C.); celottostefano@gmail.com (S.C.); marinafriuli58@gmail.com (M.P.); sara.lovascio@gmail.com (S.L.); kate.u@libero.it (K.U.)

**Keywords:** herpes zoster, vaccines, adherence to vaccination, vascular events

## Abstract

**Background/Objectives**: Recently, the Herpes Zoster (HZ) vaccination has been introduced, alongside influenza and pneumococcal vaccination, at age 65. Factors influencing adherence to this vaccination and its clinical benefits are not completely understood. The aim of this study was to evaluate factors influencing adherence to HZ vaccination compared to pneumococcal and influenza and to assess its clinical effect in preventing acute vascular events. **Methods:** A total of 1152 patients (520 males), having a birth cohort from 1952 to 1959 inclusive, was recruited, belonging to the District of Udine (N = 839) and to the ASAPs 2 and 3 of Pordenone (N = 313). For each patient, a form was compiled. **Results:** HZ vaccination was administered to 498 patients, influenza to 665, and pneumococcal to 742 (*p* < 0.0001). Among the vaccinated, 266 received the live-attenuated version, and 232 the recombinant HZ vaccine. In logistic regression, the presence of addictions, low educational level, and poor socioeconomic status were strongly associated with lower vaccine adherence. The presence of chronic diseases enhanced only pneumococcal (*p* < 0.001) and influenza (*p* < 0.001) vaccine adherence. Forty-two non-fatal acute vascular events were recorded from age 65 onwards: 14 cardiac, 20 cerebrovascular, and 8 peripheric. Only 6/493 patients experienced an event following HZ vaccination compared to 36/659 unvaccinated subjects (*p* = 0.0003). In Cox modeling, HZ vaccination proved to be an independent predictor in preventing subsequent acute vascular events (*p* < 0.001). **Conclusions:** The presence of pathologies does not enhance adherence to HZ vaccination while an unfavorable socio-environmental context greatly hinders it. HZ vaccination, but not influenza and pneumococcal vaccination, appears to protect against the occurrence of acute vascular events.

## 1. Introduction

Health authorities in Italy recommend three vaccinations at age 65 for the entire population, regardless of health status: influenza (to be repeated annually), pneumococcal, and Herpes Zoster (HZ) vaccination, the latter regardless of whether a patient has previously contracted the disease. In the Friuli Venezia Giulia Region, the first two are performed mainly by general practitioners (GPs), while the third is administered exclusively by Prevention Departments. Determining the optimal age for influenza vaccination is particularly complex, as both individual and collective factors, as well as clinical and cost-effectiveness evaluations, must be considered [1,2]. Among individual factors, the risk of complications and/or hospitalization following influenza is very important. The Ministry of Health in Italy, in agreement with the World Health Organization, recommended annual influenza and pneumococcal vaccination starting from the age of 65 [3]. The Autonomous Region of Friuli Venezia Giulia, with Resolution No. 365 of 3 March 2017, “Update and extension of vaccination offerings for adults, elderly people and at-risk subjects in the Friuli Venezia Giulia Region,” extended HZ vaccination to those reaching the age of 65 starting in 2017.

Much is known about influenza and pneumococcal vaccinations and their protective role, especially in fragile patients, particularly those with chronic conditions. Less is known about HZ vaccination, primarily because the pathogenic role of Varicella-Zoster infection in relation to various clinical conditions is not well understood [4,5,6]. Indeed, beyond the classic skin eruption, with or without post-herpetic neuralgia, data are emerging in the literature linking this disease to an increased risk of acute vascular events such as stroke and myocardial infarction [7,8,9,10,11], particularly in the first year following infection [7,12]. This association may be mediated by VZV-associated exosomes that release clotting factors into the bloodstream [13,14] or by VZV replication in arterial walls resulting in cerebral vasculopathy [15]. Vaccination and antiviral treatment may mitigate the HZ-associated vascular risk [16]. Therefore, HZ vaccination has acquired a significance not previously attributable. However, is this reflected in clinical practice, or does vaccine hesitancy prevail for this vaccination?

Vaccine hesitancy has been included in the list of ten threats to global health [17]. The WHO definition of vaccine hesitancy is as follows: “Vaccine hesitancy refers to delay in acceptance or refusal of vaccines despite availability of vaccination services. Vaccine hesitancy is complex and context-specific, varying across time, place, and vaccines. It is influenced by factors such as complacency, convenience, and confidence” [18]. To simplify, we could say that any subject who, despite having the opportunity to vaccinate themselves or their children, refuses, delays, fears, or tries to reschedule established doses is defined as hesitant [19]. This definition encompasses a wide range of behaviors, even very different from each other, but all sharing a single result: lack of adherence to vaccination campaigns [20]. Various factors determine vaccine hesitancy: contextual determinants, individual and/or group determinants, and those related to a vaccine or vaccination [21]. The first includes historical, social, cultural, environmental, economic, political, and institutional factors that can influence vaccination choices in the population [22]. The second includes social norms and pressure from family and friends as determinants of vaccine hesitancy. Finally, elements related to the individual vaccine are certainly applicable to our context. HZ vaccination was introduced only a few years ago in our healthcare system and therefore remains partially unknown to the population. Moreover, it is administered by invitation only from Prevention Departments, unlike influenza and pneumococcal vaccinations performed mainly by GPs, whose role is fundamental in increasing vaccine adherence, especially in at-risk categories [23,24].

However, several elements are not well clarified in the literature: (a) which factors, both socio-demographic and clinical, influence adherence to HZ vaccination compared to influenza and pneumococcal vaccinations; (b) whether the elements influencing vaccine hesitancy in question are invariable or dependent on the reference territory; (c) what the clinical effects of HZ vaccination are, not only in disease prevention but also in relation to possible protective effects on the occurrence of acute vascular events. 

The aims of this study were the following:

1. To evaluate adherence to HZ vaccination compared to adherence to pneumococcal and influenza vaccinations in two geographically and socio-environmentally different cohorts: (a) urban District of Udine and (b) experimental primary care outpatient clinics (ASAP) “Dolomiti Friulane” in the foothill/mountain area of Pordenone province in Italy.
2. To identify factors most capable of influencing adherence or hesitancy to the various types of vaccines proposed to the population at age 65 years and beyond.
3. To evaluate the effect of HZ vaccination in preventing both the disease and acute vascular events.

## 2. Materials and Methods

### 2.1. Study Design

This is a retrospective longitudinal and cross-sectional cohort study.

### 2.2. Patient Recruitment

Five GPs from the Health District of Udine, Italy of the Azienda Sanitaria Universitaria Friuli Centrale (ASUFC) participated in this study: one male and four females. All had the maximum number of patients, in three cases self-limited to 1300 subjects. Data from patients of these five physicians were compared to those of subjects belonging to two ASAPs, ASAP “Dolomiti Friulane” 2 and 3 of the Azienda Sanitaria Friuli Occidentale (ASFO) Pordenone where the trainee worked. ASAP is a general medicine clinic managed by three or four doctors, in most cases trainees in family medicine, with an out-of-hours care contract but functioning as a GP. These experimental clinics were established to assist citizens who, following the cessation of their own GP, were left without the possibility of choosing a new doctor. The patient can request to be assisted by ASAP and therefore the relationship is with the clinic, not with the individual doctor. The service operates during daytime hours and if possible, is organized at the facility designated to host the Out-of-Hours Care Service.

Patient recruitment occurred in the first six months of 2025. Each participating physician from the District of Udine was asked to extract, using the software attached to the medical record, the complete list of patients in their care belonging to birth cohorts from 1952 to 1959 inclusive. “In care” means they were patients during the six months spanning 2024 and 2025. Based on this list, the trainee and thesis supervisor, in collaboration with participating physicians, compiled patient forms by drawing data from individual physicians’ management systems and from the Continuity of Care portal of the Friuli Venezia Giulia region. In the two ASFO ASAPs, the trainee extracted from the Continuity of Care portal the list of patients belonging to the aforementioned birth cohorts who had therefore turned 65 years between 2017 and 2024. For these patients, the same form was completed, integrating data through telephone contact.

### 2.3. Patient Form

The patient form was divided into thirteen sections:

1. Date of compilation.
2. Details of participating physicians: surname, name, identification code, gender, and age.
3. Patient details: origin code, surname and name, gender, date of birth, age, ethnicity (Caucasian/other), height, weight, body mass index (kg/m^2^), smoking history (no/former/current), and history of significant alcohol consumption (absent/present).
4. Social status: family composition (alone/with others), work/economic capacity (absent/present), education (elementary-middle school/high school-university), physical activity (absent/present), civil disability (absent/present), and institutionalization (absent/present).
5. Previous HZ disease (absent/present/present affecting ophthalmic nerve) and date of any infection with patient’s age.
6. HZ vaccination: absent/present.
7. HZ vaccination present: date of administration, patient’s age at vaccination, vaccine type (attenuated/recombinant/recombinant doses), participation (by invitation/autonomous), and if vaccinated, eventual facilitating role of GP (absent/present).
8. Influenza vaccination: performed at age 65 years (no/yes), concurrent with HZ vaccination (no/yes), and number of influenza vaccine doses from age 65 to end of follow-up (maximum calculable 6).
9. Pneumococcal vaccination: performed at age 65 (no/yes), concurrent with HZ vaccination (no/yes), performed from age 65 to end of follow-up (no/yes).
10. Concurrent pathologies present in the patient: arterial hypertension (absent/present), cardiovascular (absent/present), diabetes mellitus (absent/present without insulin therapy/present with insulin therapy), chronic respiratory (absent/present), chronic liver disease (absent/present), chronic kidney disease (absent/present non-dialysis/present with dialysis), oncological (absent/present), hematological–oncological (absent/present), chromic neurological diseases (absent/present), neurovascular (absent/present), mental illness (absent/anxiety–depression/psychosis), immunosuppression (absent/present), HIV (absent/present), awaiting organ or stem cell transplant (absent/present), and severe-recurrent HZ (absent/present) *.
11. Presence of non-fatal acute vascular events from age 65 to recruitment date: cardiac (absent/present), date and age at the event, cerebral (absent/present), date and age at the event, and peripheral (absent/present), date and age at the event. Acute cardiac events were predominantly ischemic heart disease, cerebral events were acute cerebrovascular events, and peripheral events were obliterative arteriopathy.

Some clarifications regarding data collection:

(a) Comprehensive evaluation of each patient, particularly recording of vascular events, was performed considering data present in the medical record at recruitment.
(b) Patient age was calculated considering the recruitment date.
(c) For analysis purposes, influenza vaccination was considered as performed if the patient had received at least 50% of theoretically available vaccinations from age 65 onwards. To avoid excessive heterogeneity, for patients with 7 or 8 years of follow up (birth cohorts 1952 and 1953), a maximum theoretical interval of 6 years was considered.
(d) Pneumococcal vaccination performed from age 65 (including years immediately preceding age 65) to end of follow-up was considered as performed.
(e) HZ vaccination was considered performed by invitation if conducted in the year of turning 65 or in the first months of the following year. In the other cases, HZ vaccination was considered autonomous.
(f) Influenza or pneumococcal vaccination was considered concurrent with HZ vaccination when all were performed at age 65 or early in the following year.
(g) The last three pathologies listed in point 10 marked with * provided no positive cases and were therefore excluded from further analysis.

### 2.4. Database

Data contained in forms were used to construct a database. Each row represented a patient and each column a variable. In constructing a patient’s database, surname and name were omitted and replaced with a progressive code for each participating physician. Once the database was completed, patient forms remained in possession of the individual participating GP responsible for the patient.

### 2.5. Statistical Analysis

Statistical analysis was performed using the following software: BMDP Release 7, STATA-SE-19, and MEDCALC 23.3.7. Categorical variables were expressed as absolute numbers and percentages, and continuous variables as median and interquartile range. Association between categorical variables was performed using Chi-square test (for linear trend when appropriate). The Mantel–Haenszel test was used to compare odds ratios from Chi-square tests. Stepwise logistic regression was used to independently identify predictive variables of vaccine adherence. Time-to-event analysis with the Mantel–Cox test was used to identify and quantify the protective role of vaccinations both in relation to the specific disease and occurrence of acute vascular events. Cox proportional hazards model was used to identify factors capable of independently predicting the occurrence of acute vascular events. For sensitivity analysis purposes, to estimate the effect of HZ vaccination on occurrence of acute vascular events considering all covariates, a HZ occurrence between age 60 and the vascular event or end of observation was considered relevant. Analysis was performed using two methods: AIPW (Augmented Inverse-Probability Weighting) using Lasso algorithm and causal mediation analysis. In AIPW using the Lasso algorithm, confounding variables are selected before evaluating causal inferences. Demographic and socio-environmental variables were entered as treatment (vaccination) covariates and clinical variables as outcome (event) covariates. Causal mediation analysis was performed to evaluate total, direct, and indirect (mediated by disease prevention) effects of HZ vaccination on the subsequent occurrence of acute vascular events.

## 3. Results

### 3.1. Recruited Population of the District of Udine and of ASAPs “Dolomiti Friulane” 2 and 3

A total of 1152 patients were recruited as follows: 520 males (45.1%), 632 females (54.9%), median age (interquartile range) 69 (67–71) years, belonging to the District of Udine (ASUFC) (N = 839, 72.8%) and to ASAPs “Dolomiti Friulane” 2 and 3 (ASFO) (N = 313, 27.2%).

Table 1 reports demographic, socio-environmental, and clinical characteristics of patients, overall and in the two cohorts separately. Between the two groups, there was no difference in gender, age, ethnicity, or body mass index. In ASAPs, a higher frequency of active smokers and people with significant alcohol consumption was found, particularly concurrent presence of both addictions. Regarding socio-environmental context, ASAPs showed lower education; regarding economic resources, perceived indigence appeared less frequently in ASAPs compared to the District of Udine. Concerning clinical characteristics, in the District of Udine a higher prevalence of liver disease, neurological disorders, and psychiatric problems such as anxiety and depression was observed.

### 3.2. Higher Adherence to Pneumococcal and Influenza than HZ Vaccination

**Figure 1:**
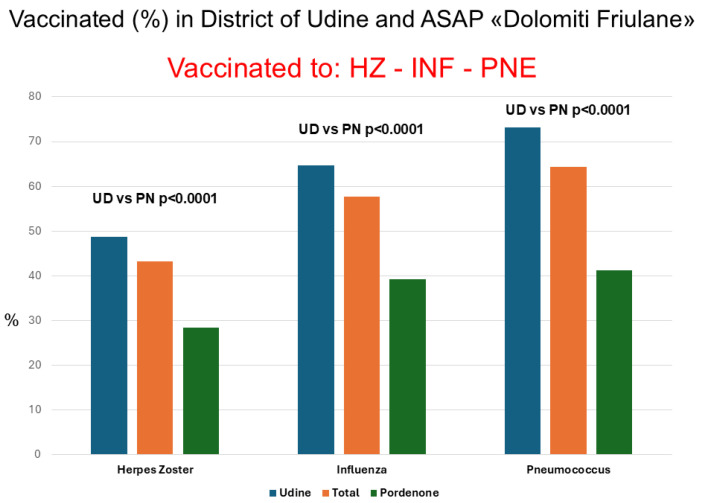
Percentage of vaccinated individuals for Herpes Zoster (HZ), influenza (INF), and pneumococcus (PNE) in the entire population and separately in the Udine District (UD) and in the Dolomiti Friulane ASAP (PN). A significant incremental linear trend in vaccination adherence was observed starting from HZ, then INF, and finally PNE both overall (*p* < 0.0001) and in the two cohorts (UD, *p* < 0.0001) and (PN, *p* < 0.0001). Vaccination adherence for all three vaccinations was significantly higher in UD compared to PN.

HZ vaccination was administered to 498 patients (43.2%), influenza vaccination to 665 (57.7%), and pneumococcal vaccination to 742 (64.4%) with a significant incremental linear trend (*p* < 0.0001, Figure 1). This pattern was confirmed both in the Udine population (*p* < 0.0001) and Pordenone population (*p* = 0.0009). Vaccine adherence was consistently significantly higher in the District of Udine compared to Pordenone ASAPs. Regarding HZ vaccination, of 498 vaccinated patients, 266 (53.4%) received the live-attenuated vaccine and 232 (46.6%) the recombinant vaccine. In 267 (53.6%) cases, patients received vaccination autonomously, while 231 (46.4%) subjects previously consulted their GP to decide. In the District of Udine, 225/409 (55.0%) vaccinated patients first contacted their GP, while this occurred in only 6/89 (6.7%) subjects belonging to ASAPs (*p* < 0.0001).

### 3.3. Factors Influencing Adherence to HZ Pneumococcal and Influenza Vaccination

Table 2, Table 3 and Table 4 report associations between the three vaccinations (HZ, pneumococcus, and influenza) and all socio-demographic and clinical variables considered in the study. Data are reported both for the general population and separately for Udine and Pordenone.

**Figure 2:**
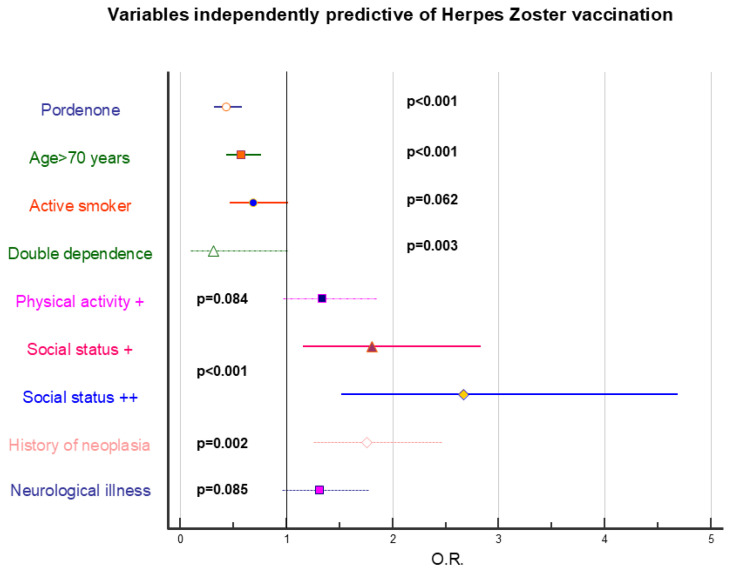
Forest plot showing the odds ratios, 95% confidence limits, and significance levels of variables identified as independently predictive of Herpes Zoster vaccination uptake. Statistical analysis was performed using stepwise logistic regression. The variables included in the analysis are those reported in Table 2. +: fairly positive context; ++: very positive context.

**Figure 3:**
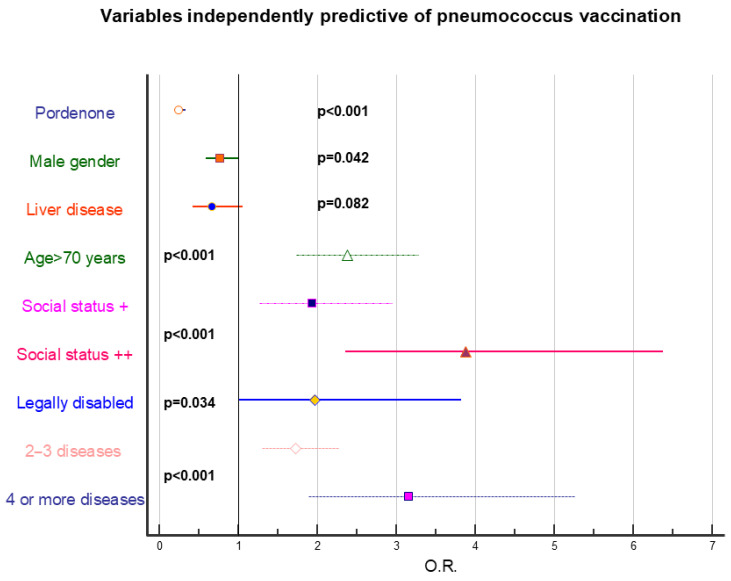
Forest plot showing the odds ratios, 95% confidence limits, and significance levels of variables identified as independently predictive of pneumococcal vaccination uptake. Statistical analysis was performed using stepwise logistic regression. The variables included in the analysis are those reported in Table 3. +: fairly positive context; ++: very positive context.

**Figure 4:**
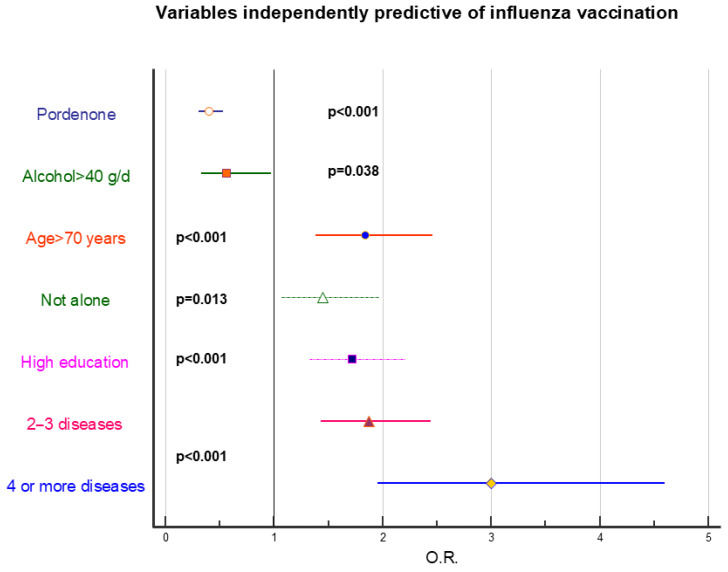
Forest plot showing the odds ratios, 95% confidence limits, and significance levels of variables identified as independently predictive of influenza vaccination uptake (at least 50% of theoretically available vaccinations over the years). Statistical analysis was performed using stepwise logistic regression. The variables included in the analysis are those reported in Table 4.

Figure 2, Figure 3 and Figure 4 represent stepwise logistic regression data performed to identify independently predictive factors for HZ, pneumococcal, and influenza vaccination. Presence of addictions was invariably associated with lower vaccine adherence, regardless of vaccination type, and a negative role was also exerted by low education and generally poor socioeconomic status. Presence of pathologies strongly positively conditionate vaccine adherence for pneumococcal and influenza vaccination; conversely, it has a marginal role regarding HZ vaccination.

### 3.4. HZ Vaccination Prevents HZ Disease

**Figure 5:**
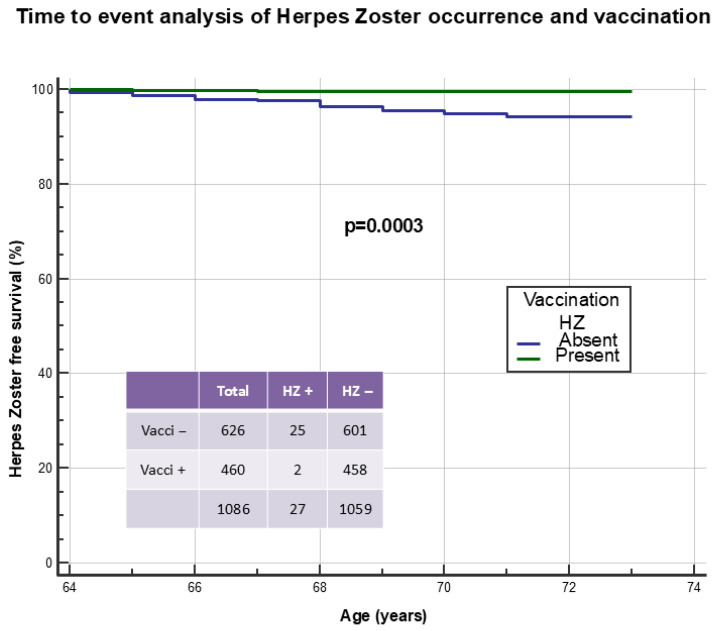
Time-to-event analysis to identify the effect of Herpes Zoster vaccination in preventing Zoster disease. Cases of Zoster occurring before 65 years of age were excluded from the analysis (N = 66).

HZ disease was found in 93 cases (8.1%): in the District of Udine in 58 (6.9%) cases (7 of the ophthalmic branch of trigeminal nerve), and in ASAPs in 35 (11.2%) cases (5 of the ophthalmic branch) (*p* = 0.018). In two cases (2.1%), HZ disease occurred after HZ vaccination; in the remaining 91 cases, in 33 (35.5%), HZ disease occurred before age 65 without subsequent vaccination, in 33 (35.5%) before age 65 followed by vaccination over time, in 20 cases (21.5%) from age 65 onwards without subsequent vaccination, and finally in 5 (5.4%) from age 65 with subsequent vaccination. No significant difference was found between the prevalence of this vaccination in patients without or with HZ previous disease (458/1059, 43.2% vs. 38/93, 40.9%, *p* = 0.656). Figure 5 illustrates a time-to-event analysis of HZ vaccination in preventing subsequent disease, excluding 66 cases who presented it before age 65. Only 2/460 (0.4%) patients presented HZ following vaccination, while conversely this occurred in 25/626 (4.0%) subjects not vaccinated for this disease (*p* = 0.0003). Results were confirmed considering all patients: 2/460 (0.4%) HZ following vaccination vs. 91/692 (13.2%) HZ before vaccination (N = 38) or HZ in never vaccinated (N = 53) (*p* < 0.0001).

### 3.5. HZ Vaccination Prevents Acute Vascular Events

Forty-two non-fatal acute vascular events were recorded from age 65 onwards: 14 cardiac (33.3%), 20 cerebrovascular (47.6%), 7 peripheral (16.7%), and 1 (2.4%) concurrently cardiac and peripheral. Events were more frequent in ASAPs 18/313 (5.8%) compared to District of Udine 24/839 (2.9%) (*p* = 0.020). In one patient (2.4%), the event (cardiac) was preceded by HZ, which occurred seven months earlier, affecting the ophthalmic branch of the trigeminal nerve. In another patient (2.4%), HZ occurred about three years after a cerebral vascular event. Table 5 illustrates associations between socio-demographic and clinical variables and event occurrence. Strong association was found with older age and male gender, as well as with arterial hypertension and multi-morbidity.

**Figure 6:**
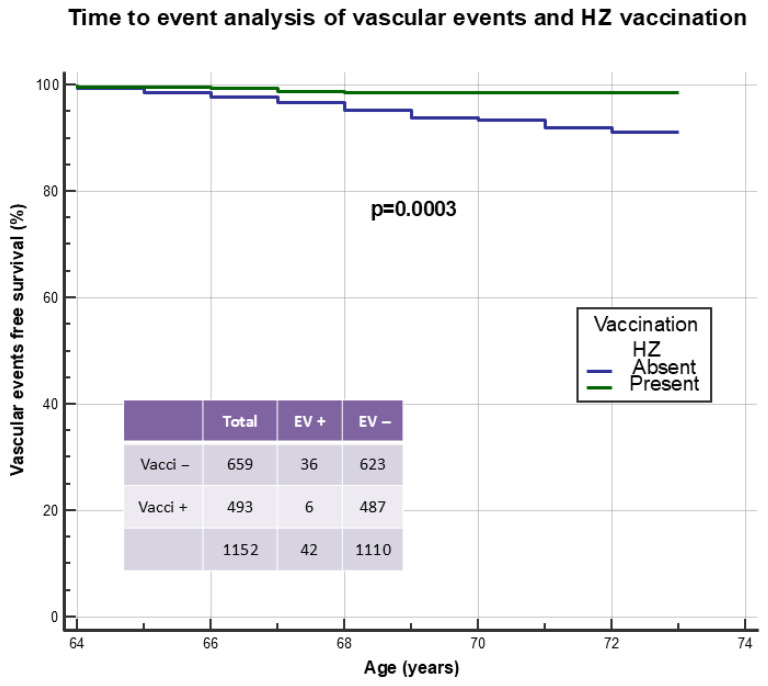
Time-to-event analysis to identify the effect of Herpes Zoster vaccination in preventing the occurrence of acute vascular events. The five patients in whom anti-Herpes Zoster vaccination had been performed after the occurrence of the event were included in the unvaccinated group.

Figure 6 illustrates time-to-event analysis of HZ vaccination in preventing acute vascular events during the observation period from age 65 onwards. In five cases, vaccination was performed after the event and these patients were included among the unvaccinated. Only 6/493 (1.2%) patients experienced an event following vaccination compared to 36/659 (5.5%) unvaccinated subjects (*p* = 0.0003). Time-to-event analysis was also constructed, distributing patients as follows: unvaccinated patients (A: N = 659), patients vaccinated with live-attenuated vaccine (B: N = 266), and finally patients vaccinated with recombinant vaccine (C: N = 227). Acute vascular events were distributed as follows: group A 36/659 (5.5%), group B 5/266 (1.9%), and group C 1/227 (0.4%, *p* = 0.0005 for linear trend). Table 6 reports variables that were independent predictors of acute vascular events in Cox proportional hazards model. All variables reported in Table 5 were used as covariates, in addition to HZ disease and related vaccination. The independent predictive role of HZ vaccination in preventing subsequent acute vascular events was evident.

Time-to-event analysis was also performed to test a possible protective role against acute vascular events for both influenza and pneumococcal vaccination, without obtaining significant results: influenza vaccination (≥50% of the theoretical doses) events in vaccinated (24/665) vs. unvaccinated (18/487, *p* = 0.556), influenza vaccination (at age 65) events in vaccinated (20/575) vs. unvaccinated (22/577, *p* = 0.746), pneumococcal vaccination (≥1 dose) events in vaccinated (24/742) vs. unvaccinated (18/410, *p* = 0.105), and pneumococcal vaccination (at age 65) events in vaccinated (14/532) vs. unvaccinated (28/620, *p* = 0.139).

For sensitivity analyses, an HZ episode occurring between age 60 and the vascular event or end of observation was considered relevant (N = 61, 5.3%). AIPW with the Lasso algorithm showed that HZ vaccination (covariates: demographic and socio-environmental variables from Table 5) was strongly protective against occurrence of acute vascular events (covariates: clinical variables from Table 5 and HZ disease) (coefficient: −0.042, standard error: 0.010, *p* < 0.001). Causal mediation analysis showed a strong direct effect (coefficient: −0.043, standard error: 0.011, *p* < 0.001) of HZ vaccination in preventing vascular events; the indirect effect mediated by HZ prevention was lower but still within the range of significance (coefficient: −0.001, standard error: 0.000, *p* = 0.025).

## 4. Discussion

Vaccinations are part of the major chapter of preventive medicine, which aims to prevent diseases in the population. Traditional medicine focuses on improving health through identification and treatment of health disorders that have already produced symptoms or complications. Conversely, preventive medicine aims to prevent the onset of pathological conditions, also focusing on diagnosing problems before symptoms or complications onset, when chances of recovery are maximum. If provided at appropriate times and in appropriate ways, prevention improves general health conditions and reduces healthcare costs. Starting from this concept, we wanted to evaluate how important the environment, socio-healthcare and care context, as well as clinical conditions, can be in influencing a well-known and widespread preventive medicine practice such as vaccination practice in adults/elderly starting from age 65 years.

### 4.1. Higher Adherence to Pneumococcal and Influenza than HZ Vaccination

Vaccination against HZ is able to very effectively reduce the risk of developing HZ and post-herpetic neuralgia (one of the most frequent and debilitating complications of the disease) [25,26]. This finding was confirmed in our study: of 27 subjects with history of HZ from age 65 onwards, only 2 had been previously vaccinated, while 25 had not received the vaccine. For HZ vaccination, 1 or 2 doses are provided, depending on the vaccine used, to be offered annually to the cohort of sixty-five-year-olds. According to regional health authority indications, this type of vaccination is primarily aimed at all people aged 65 and over starting with those born in 1952; secondly, at people over 18 years with the following pathologies: chronic heart disease (excluding isolated arterial hypertension), diabetes mellitus [27], chronic pulmonary pathologies, chronic obstructive pulmonary disease, people awaiting or undergoing immunosuppressive therapy or dialysis, people awaiting or transplanted with solid organ or hematopoietic stem cell transplantation (bone marrow), oncological and onco-hematological pathologies, HIV positivity, single complicated episode of HZ (post-herpetic neuralgia and/or encephalitis, ophthalmic and/or auricular herpes zoster), and HZ recurrences (at least two episodes certified by specialist or attending physician) [25,28,29,30,31,32,33]. It is evident that chronic pathologies highlighted in this list should be an additional indication to perform HZ vaccination even after the age of 65 years. Unfortunately, this did not occur at all, either in the Udine cohort or in the Pordenone cohort. Conversely, socio-environmental factors such as low education level and/or low attention to one’s health status, better if in combination with each other and with other elements suggestive of social discomfort, seem to have an almost exclusive role in negatively conditioning adherence to this vaccination.

What is the possible explanation for this unsatisfactory result when measured against objectives to be pursued? The simplest answer appears linked to the GP’s role in relation to HZ vaccination. Indeed, in Udine, greater adherence was obtained in relation to the presence of pro-vaccination suggestion and advice from the GP compared to what occurred in the Pordenone cohort, but this seems to modify the quantity (prevalence) of the result, not its quality (vaccination of the fragile). Administration of this vaccine exclusively by Prevention Departments of the Friuli Venezia Giulia Region would seem to hinder greater vaccination efficiency. On the other hand, the GP has ample potential in the vaccination field for several reasons: (a) fiduciary relationship based on free choice with their patient, (b) scientific and cultural training that also confers managerial skills, and (c) position within the National Health Service as first approach and guarantor of primary care needs [34]. In practice, the GP manages to optimize contexts by seizing and exploiting even small opportunities for dialogue on this topic with the patient.

In our region, the foothill and mountain areas, both in Udine and Pordenone provinces, represent partially disadvantaged districts, with fewer environmental opportunities, inhabited by people with, on average, fewer economic resources, as shown by income tax return reports. Conversely, the District of Udine, and particularly its northern area, represents a dynamic, evolving territory with good resources and likely higher average cultural level. Therefore, comparing vaccine adherence in these two types of populations could represent a good model, also exportable to other contexts. Indeed, characteristics of territories of Dolomiti Friulane ASAPs in Pordenone province proved different from those of the District of Udine, especially in degree of smoking and alcohol addiction, which were higher in Pordenone ASAPs, and in education level, which was significantly higher in the District of Udine. Comparison of health status between the two populations reserved surprises that perhaps merit a comment. Paradoxically, the District of Udine population would appear sicker and affected by a greater number of pathologies compared to the ASAP population. Indeed, in Udine, a greater number of liver diseases, neurological diseases in the most general sense, and especially higher incidence of anxiety and depression were recorded. Apart from anxiety and depression and other neurological disorders such as the headaches that accompany more evolved societies compared to less stressful ones, it appears difficult that in a context characterized by higher alcohol consumption, the number of liver diseases would be less relevant. Also, this may be due to the fact that the number of acute vascular events from age 65 onwards was significantly higher in ASAP subjects compared to Udine residents. Suspicion arises that active and proactive attention to health problems in marginal contexts drops sharply, only to then record a greater number of acute events.

### 4.2. Factors Influencing Adherence to HZ Pneumococcal and Influenza Vaccination

Pneumococcal and influenza vaccination have been part of work normally performed by GPs for years, constituting a significant part of their professional commitment. Indeed, in relation to this fundamental element, they differ in many respects from Herpes vaccination. Evaluating adherence to these two vaccinations was not as simple as for the Herpes Zoster. For Pneumococcus, any vaccination performed was considered an expression of adherence, even if, in some cases, it was performed in years preceding age 65, and in any case disregarding the fact that occasionally two vaccinations were performed. For influenza vaccination, years elapsed from age 65 to the study time also had to be considered. Indeed, can a person who receives an influenza vaccination only at age 65 and then for all remaining years up to age 72 no longer vaccinates be considered adherent? Therefore, a percentage ratio was constructed with denominator being number of years elapsed by patient from age 65 to end of follow-up (maximum 6 years) and with numerator being number of influenza vaccinations performed. For birth cohorts 1952 and 1953, the entire elapsed time (7–8 years) was not considered, but the denominator was brought to 6 years, to avoid creating too great heterogeneity compared to cohorts born in subsequent years. A value ≥50% of the ratio was considered the expression of vaccine adherence for influenza.

Adherence to these vaccinations (influenza and pneumococcus), although still greater in the Udine cohort compared to Pordenone, was significantly higher than that for HZ. Pneumococcal vaccination appeared to have found the greatest acceptance among the population. Yet most importantly, influenza and pneumococcal vaccination were independently associated with patient clinical status, meaning greater vaccine adherence in patients with common chronic cardio-circulatory, respiratory pathologies, and diabetes, especially if co-present. The negative role of a less favorable socio-environmental status did not disappear, but the GP still managed to vaccinate a portion of subjects a priori unlikely to vaccinate. This was at least in the Udine context, while unfortunately, in Pordenone, low vaccine adherence was associated with irrelevance of clinical picture in relation to vaccine adherence. It is likely that in these areas, even before the current situation in which presence of ASAPs certifies non-existence of the traditional GP figure, there may have been a deficiency in the presence of the family doctor figure with cascading the situation photographed by this work.

Previous HZ disease was found in 93 patients with prevalence therefore below 10%. Calculating the exact prevalence of this disease in the population appears particularly complicated [29,35]. It is estimated that during their lifetime, about 25% of subjects have an episode of HZ. Certainly, incidence increases with age, and doubles from subjects under 50 years to subjects over 70 years of age. Even considering our patients’ age, the prevalence found does not differ particularly from the real one, although some data loss cannot be excluded since HZ is a pathology that in most cases resolves. Regarding localization at the ophthalmic branch of trigeminal nerve, the obtained prevalence of about 13% is perfectly in line with the literature data [36,37,38].

### 4.3. HZ Vaccination Prevents Acute Vascular Events

However, what proved most surprising and interesting was the finding that HZ vaccination exerted a protective effect against development of acute vascular events, cardiac, cerebral, and peripheral. In particular, the protective effect would appear more evident for recombinant vaccine compared to live-attenuated vaccine, although a limiting factor could be represented by the fact that recombinant vaccine was introduced into practice after the live-attenuated one, and is therefore associated with shorter follow-up. In recent years, great relevance has been acquired by the observation, previously little known, of an association between HZ disease and acute vascular events [8,9,10,11,39,40,41,42], particularly cerebrovascular and cardiovascular [7,16,43,44,45,46]. Consistent with this observation would be evidence of a protective effect of HZ vaccination against occurrence over time of these acute events, particularly stroke [12,47,48,49,50]. This effect would appear to be exerted independently of detection of possible subsequent HZ infection in unvaccinated individuals [51] and type of vaccine used [48,51,52]. In parallel, data from very recent literature would suggest a protective role of HZ vaccination against the development of dementia, particularly vascular dementia [53,54,55,56,57,58,59]. Interpretation of the possible protective effect of HZ vaccination on occurrence of acute vascular events is particularly intriguing, especially in the absence of a recent episode of HZ disease. Varicella-Zoster virus infection in the past, before advent of vaccination, affected large segments of the population. Its latency in the host organism is characterized by reactivations that may also be subclinical [6], which in turn could have a causal role, through an inflammatory mechanism, in genesis of vascular damage [60]; this is regardless of occurrence of a frank episode of HZ [42]. Therefore, a protective role of vaccination in this context cannot be excluded [61,62,63]. What was observed could be affected by confounding variables such as better clinical condition in vaccinated patients compared to unvaccinated. However, although not all possible health variables were evaluated, regarding what was analyzed in this study, health status between vaccinated and unvaccinated did not differ, at least regarding the cardiovascular aspect. Certainly, unvaccinated patients had riskier behavior and less favorable social context than vaccinated ones. However, our finding was confirmed by multivariate analysis using Cox proportional hazards model and by two sensitivity analyses performed. It should finally be noted that such findings were not replicated considering both influenza and pneumococcal vaccination.

The present work has limitations. First, it is a retrospective work with limitations inherent to this type of design. A prospective observational study would be more accurate and effective. The most interesting aspect of this work concerns the protective effect of HZ vaccination against the occurrence of acute vascular events. Calculating the sample size necessary to obtain solid results is not easy. We can assume that in the cohort of patients aged 65 to 72 years, with a mean age of approximately 68.6 years, the overall incidence of cerebrovascular, cardiovascular, and peripheral events is 3.9% per year [64]. Considering an average follow-up of 3.4 years, we obtain a cumulative incidence of 12.4%. Calculating a relative risk reduction of 23% [52] and an alpha error of 0.05, a total of about 3800 patients will be necessary to achieve a result with a power of 80%. Therefore, our data appears to be suggestive but likely, in this context, not conclusive. Sample size could have been expanded, to obtain a greater number of events and higher statistical power, but this would have required a longer period for data collection. Longer follow-up could have implemented results in terms of events. However, this was not possible in relation to the study design that includes patients who turned 65 starting from 2017, the year in which Friuli Venezia Giulia Region introduced HZ, offering vaccination to sixty-five-year-olds. Recording of some variables, such as lipid profile, could have better defined cardiovascular risk in these patients. Finally, as defined in methods, patients who were in care of the GP at recruitment time were included. This fact may have resulted in failure to include some patients who died in previous years; however, it would have been difficult to recover vaccination data of deceased persons since they were no longer present in the region’s portal Continuity of Care. In any case, the data seems interesting, obtained moreover in the general practice setting that allows more accurate collection of clinical data compared to other contexts.

## 5. Conclusions

Having contracted HZ previously does not appear to influence adherence to HZ vaccination. Conversely, an unfavorable socio-environmental context plays a primary role in hindering vaccine adherence. Presence of pathologies such as cardio-circulatory, chronic respiratory, and diabetes mellitus plays a very important role in favoring vaccine adherence toward influenza and pneumococcal vaccination, at least in non-marginalized contexts and in presence of a GP. HZ vaccination, but not influenza and pneumococcal vaccination, appears to exert a protective role against occurrence of cardiac, cerebral, and peripheral acute vascular events.

## Figures and Tables

**Table 1:** Socio-demographic and clinical characteristics of the studied population (N = 1152). Data are presented as a total and separately for Udine and Pordenone. The percentages apply to the column total. Statistical analysis was performed by means of the Chi-square test.

Socio-Demographic and Clinical Variables	Total N = 1152		Udine N = 839	Pordenone N = 313	*p*
	N	%			
Male gender	520	45.1	368 (43.9%)	152 (48.6%)	0.154
Age > 70 years	312	27.1	222 (26.5%)	90 (28.8%)	0.436
Non-Caucasian ethnicity	9	0.8	4 (0.5%)	5 (1.6%)	0.055
BMI > 30 kg/m^2^	184	16.0	132 (15.7%)	52 (16.6%)	0.717
Active smoker *	171	14.8	107 (12.8%)	64 (20.4%)	0.001
Alcohol intake > 40 g/d	63	5.5	36 (4.3%)	27 (8.6%)	0.004
Double dependence	27	2.3	13 (1.5%)	14 (4.5%)	0.003
Not living alone	905	78.6	654 (77.9%)	251 (80.2%)	0.409
Sufficient economic resources	1100	95.5	792 (94.4%)	308 (98.4%)	0.004
High educational level	574	49.8	455 (54.2%)	119 (38.0%)	<0.0001
Physical activity present	542	47.0	373 (44.5%)	169 (54.0%)	0.004
Total deficiencies ≤ 1	685	59.5	501 (59.7%)	184 (58.8%)	0.775
Legal disability	64	5.6	45 (5.4%)	19 (6.1%)	0.641
Institutionalized	5	0.4	2 (0.2%)	3 (1.0%)	0.098
Arterial hypertension	614	53.3	433 (51.6%)	181 (57.8%)	0.060
Cardiovascular disease	304	26.4	222 (26.5%)	82 (26.2%)	0.928
Diabetes mellitus	138	12.0	93 (11.1%)	45 (14.4%)	0.126
Chronic respiratory	134	11.6	102 (12.2%)	32 (10.2%)	0.362
Chronic liver disease	121	10.5	106 (12.6%)	15 (4.8%)	0.0001
Chronic nephropathy	41	3.6	35 (4.2%)	6 (1.9%)	0.066
Oncological	183	15.9	141 (16.8%)	42 (13.4%)	0.162
Haemato-oncological	19	1.6	16 (1.9%)	3 (1.0%)	0.261
Neurological	237	20.6	209 (24.9%)	28 (8.9%)	<0.0001
Neurovascular	53	4.6	44 (5.2%)	9 (2.9%)	0.088
Psychiatric	290	25.2	249 (29.7%)	41 (13.1%)	<0.0001
Immuno-suppression	53	4.6	42 (5.0%)	11 (3.5%)	0.282
Total diseases > 2	339	29.4	271 (32.3%)	68 (21.7%)	0.0005
Previous Herpes Zoster	93	8.1	58 (6.9%)	35 (11.2%)	0.018

BMI = body mass index; * = 325 (28.2%) patients were past smokers.

**Table 2:** Association between Herpes Zoster vaccination and socio-demographic and clinical variables. Data are presented as a total and separately for Udine and Pordenone. The percentages apply to the rows, considering either the total or the two subgroups. Statistical analysis was performed by means of the Chi-square test. Mantel–Haenszel test ▲ was adopted to compare the odds ratio between the two groups. Variables with a reddish background were found to decrease adhesion, while those with soft green enhance it.

	Herpes Zoster Vaccination Accomplished						
Socio-Demographic and Clinical Variables	Total N = 498 (43.2%)	*p*	Udine N = 409 (48.7%)	*p*	Pordenone N = 89 (28.4%)	*p*	*p* ▲
Male gender (N = 520)	225 (43.3%)	0.980	185 (50.3%)	0.435	40 (26.3%)	0.419	0.819
Age > 70 years (N = 312)	104 (33.3%)	<0.0001	93 (41.9%)	0.017	11 (12.2%)	0.0001	0.0001
Non-Caucasian ethnicity (N = 9)	5 (55.6%)	0.454	2 (50.0%)	0.960	3 (60.0%)	0.115	0.425
BMI > 30 kg/m^2^ (N = 184)	75 (40.8%)	0.461	59 (44.7%)	0.310	16 (30.8%)	0.683	0.548
Active smoker (N = 171)	53 (31.0%)	0.0005	43 (40.2%)	0.058	10 (15.6%)	0.011	0.004
Alcohol intake > 40 g/d (N = 63)	21 (33.3%)	0.103	13 (36.1%)	0.121	8 (29.96%)	0.885	0.313
Double dependence (N = 27)	4 (14.8%)	0.002	3 (23.1%)	0.062	1 (7.1%)	0.071	0.017
Not living alone (N = 905)	413 (45.6%)	0.001	337 (51.5%)	0.002	76 (30.3%)	0.146	0.001
Economic resources + * (N = 1100)	482 (43.8%)	0.063	394 (49.7%)	0.017	88 (28.6%)	0.673	0.024
High educational level (N = 574)	286 (49.8%)	<0.0001	242 (53.2%)	0.005	44 (37.0%)	0.009	0.0003
Physical activity + ** (N = 542)	269 (49.6%)	<0.0001	210 (56.3%)	0.0001	59 (34.9%)	0.006	<0.0001
Total deficiencies ≤ 1 (N = 685)	336 (49.1%)	<0.0001	273 (54.5%)	0.0001	63 (34.2%)	0.007	<0.0001
Legal disability (N = 64)	21 (32.8%)	0.083	18 (40.0%)	0.227	3 (15.8%)	0.207	0.122
Institutionalized (N = 5)	1 (20.0%)	0.293	1 (50.0%)	0.972	0 (0.0%)	0.273	0.755
Arterial hypertension (N = 614)	270 (44.0%)	0.586	224 (51.7%)	0.074	46 (25.4%)	0.165	0.399
Cardiovascular disease (N = 304)	132 (43.4%)	0.937	117 (52.7%)	0.169	15 (18.3%)	0.018	1.000
Diabetes mellitus (N = 138)	54 (39.1%)	0.300	44 (47.3%)	0.769	10 (22.2%)	0.318	0.497
Chronic respiratory (N = 134)	56 (41.8%)	0.721	48 (47.1%)	0.716	8 (25.0%)	0.649	0.662
Chronic liver disease (N = 121)	55 (45.5%)	0.601	53 (50.0%)	0.783	2 (13.3%)	0.184	0.932
Chronic nephropathy (N = 41)	19 (46.3%)	0.682	17 (48.6%)	0.983	2 (33.3%)	0.788	1.000
Oncological (N = 183)	97 (53.0%)	0.004	80 (56.7%)	0.037	17 (40.5%)	0.063	0.009
Haemato-oncological (N = 19)	10 (52.6%)	0.404	10 (62.5%)	0.266	0 (0.0%)	0.273	0.691
Neurological (N = 237)	121 (51.1%)	0.006	113 (54.1%)	0.076	8 (28.6%)	0.987	0.110
Neurovascular (N = 53)	21 (39.6%)	0.587	19 (43.2%)	0.448	2 (22.2%)	0.675	0.473
Psychiatric (N = 290)	133 (45.9%)	0.295	121 (48.6%)	0.954	12 (29.3%)	0.899	1.000
Immuno-suppression (N = 53)	29 (54.7%)	0.084	25 (59.5%)	0.152	4 (36.4%)	0.553	0.160
Total diseases > 2 (N = 339)	161 (47.5%)	0.059	142 (52.4%)	0.144	19 (27.9%)	0.919	0.229
Previous Herpes Zoster (N = 93)	40 (43.0%)	0.965	28 (48.3%)	0.941	12 (34.3%)	0.415	0.775

BMI = body mass index; * sufficient economic resources; ** physical activity present.

**Table 3:** Association between pneumococcus vaccination and socio-demographic and clinical variables. Data are presented as a total and separately for Udine and Pordenone. The percentages apply to the rows, considering either the total or the two subgroups. Statistical analysis was performed by means of the Chi-square test. Mantel–Haenszel test ▲ was adopted to compare the odds ratio between the two groups. Variables with a reddish background were found to decrease adhesion, while those with soft green enhance it.

	Pneumococcus Vaccination Accomplished (At Least One)						
Socio-Demographic and Clinical Variables	Total N = 742 (64.4%)	*p*	Udine N = 613 (73.1%)	*p*	Pordenone N = 129 (41.2%)	*p*	*p* ▲
Male gender (N = 520)	323 (62.1%)	0.140	267 (72.6%)	0.769	56 (36.8%)	0.127	0.299
Age > 70 years (N = 312)	236 (75.6%)	<0.0001	185 (83.3%)	0.0001	51 (56.7%)	0.0004	<0.0001
Non-Caucasian ethnicity (N = 9)	6 (66.7%)	0.887	3 (75.0%)	0.930	3 (60.0%)	0.390	0.713
BMI > 30 kg/m^2^ (N = 184)	122 (66.3%)	0.558	107 (81.1%)	0.024	15 (28.8%)	0.047	0.525
Active smoker (N = 171)	97 (56.7%)	0.023	75 (70.1%)	0.458	22 (34.4%)	0.213	0.203
Alcohol intake > 40 g/d (N = 63)	33 (52.4%)	0.040	22 (61.1%)	0.098	11 (40.7%)	0.958	0.272
Double dependency (N = 27)	13 (48.1%)	0.074	8 (61.5%)	0.345	5 (35.7%)	0.669	0.462
Not living alone (N = 905)	593 (65.5%)	0.130	491 (75.1%)	0.013	102 (40.6%)	0.677	0.078
Economic resources + * (N = 1100)	715 (65.0%)	0.054	587 (74.1%)	0.005	128 (41.6%)	0.331	0.005
High educational level (N = 574)	403 (70.2%)	<0.0001	342 (75.2%)	0.135	61 (51.3%)	0.005	0.006
Physical activity + ** (N = 542)	366 (67.5%)	0.037	287 (76.9%)	0.023	79 (46.7%)	0.031	0.002
Total deficiencies ≤ 1 (N = 685)	468 (68.3%)	0.0008	384 (76.6%)	0.004	84 (45.7%)	0.057	0.0008
Legal disability (N = 64)	48 (75.0%)	0.068	41 (91.1%)	0.005	7 (36.8%)	0.689	0.057
Institutionalized (N = 5)	2 (40.0%)	0.253	2 (100%)	0.390	0 (0.0%)	0.145	0.851
Arterial hypertension (N = 614)	418 (68.1%)	0.005	337 (77.8%)	0.001	81 (44.8%)	0.136	0.0006
Cardiovascular disease (N = 304)	221 (72.7%)	0.0004	183 (82.4%)	0.0002	38 (46.3%)	0.272	0.0003
Diabetes mellitus (N = 138)	99 (71.7%)	0.055	79 (84.9%)	0.006	20 (44.4%)	0.634	0.018
Chronic respiratory (N = 134)	100 (74.6%)	0.009	90 (88.2%)	0.0002	10 (31.3%)	0.227	0.018
Chronic liver disease (N = 121)	81 (66.9%)	0.538	74 (69.8%)	0.419	7 (46.7%)	0.660	0.648
Chronic nephropathy (N = 41)	34 (82.9%)	0.012	31 (88.6%)	0.035	3 (50.0%)	0.659	0.054
Oncological (N = 183)	130 (71.0%)	0.041	110 (78.0%)	0.146	20 (47.6%)	0.365	0.105
Haemato-oncological (N = 19)	16 (84.2%)	0.069	14 (87.5%)	0.189	2 (66.7%)	0.368	0.188
Neurological (N = 237)	177 (74.7%)	0.0002	164 (78.5%)	0.042	13 (46.4%)	0.557	0.044
Neurovascular (N = 53)	38 (71.7%)	0.256	34 (77.3%)	0.518	4 (44.4%)	0.842	0.609
Psychiatric (N = 290)	208 (71.7%)	0.003	190 (76.3%)	0.169	18 (43.9%)	0.707	0.187
Immuno-suppression (N = 53)	38 (71.7%)	0.256	35 (83.3%)	0.124	3 (27.3%)	0.339	0.480
Total diseases > 2 (N = 339)	256 (75.5%)	<0.0001	222 (81.9%)	0.0001	34 (50.0%)	0.096	<0.0001
Previous Herpes Zoster (N = 93)	61 (65.6%)	0.804	44 (75.9%)	0.618	17 (48.6%)	0.348	0.386

BMI = body mass index; * sufficient economic resources; ** physical activity present.

**Table 4:** Association between influenza vaccination (at least 50% of theoretically available vaccinations over the years) and socio-demographic and clinical variables. Data are presented as a total and separately for Udine and Pordenone. The percentages apply to the rows, considering either the total or the two subgroups. Statistical analysis was performed by means of the Chi-square test. Mantel–Haenszel test ▲ was adopted to compare the odds ratio between the two groups. Variables with a reddish background were found to decrease adhesion, while those with soft green enhance it.

	Influenza Vaccination Accomplished (At Least 50%)						
Socio-Demographic and Clinical Variables	Total N = 665 (57.7%)	*p*	Udine N = 542 (64.6%)	*p*	Pordenone N = 123 (39.1%)	*p*	*p* ▲
Male gender (N = 520)	296 (56.9%)	0.617	238 (64.7%)	0.969	58 (38.2%)	0.688	0.906
Age > 70 years (N = 312)	211 (67.6%)	<0.0001	166 (74.8%)	0.0002	45 (50.0%)	0.014	<0.0001
Non-Caucasian ethnicity (N = 9)	6 (66.7%)	0.586	3 (75.0%)	0.663	3 (60.0%)	0.339	0.510
BMI > 30 kg/m^2^ (N = 184)	115 (62.5%)	0.153	96 (72.7%)	0.033	19 (36.5%)	0.656	0.142
Active smoker (N = 171)	85 (49.7%)	0.021	66 (61.7%)	0.499	19 (29.7%)	0.078	0.130
Alcohol intake > 40 g/d (N = 63)	26 (41.3%)	0.006	17 (47.2%)	0.026	9 (33.3%)	0.507	0.047
Double dependency (N = 27)	11 (40.7%)	0.071	7 (53.8%)	0.414	4 (28.6%)	0.401	0.332
Not living alone (N = 905)	537 (59.3%)	0.034	440 (67.3%)	0.002	97 (38.6%)	0.635	0.022
Economic resources + * (N = 1100)	640 (58.2%)	0.149	519 (65.5%)	0.021	121 (39.3%)	0.974	0.043
High educational level (N = 574)	374 (65.2%)	<0.0001	317 (69.7%)	0.0008	57 (47.9%)	0.015	<0.0001
Physical activity + ** (N = 542)	318 (58.7%)	0.540	250 (67.0%)	0.189	68 (40.2%)	0.712	0.213
Total deficiencies ≤ 1 (N = 685)	424 (61.9%)	0.0005	345 (68.9%)	0.002	79 (42.9%)	0.116	0.0006
Legal disability (N = 64)	43 (67.2%)	0.115	37 (82.2%)	0.011	6 (31.6%)	0.477	0.111
Institutionalized (N = 5)	3 (60.0%)	0.918	2 (100%)	0.295	1 (33.3%)	0.832	0.978
Arterial hypertension (N = 614)	382 (62.2%)	0.001	303 (70.0%)	0.0008	79 (43.6%)	0.065	0.0002
Cardiovascular disease (N = 304)	206 (67.8%)	<0.0001	171 (77.0%)	<0.0001	35 (42.7%)	0.465	<0.0001
Diabetes mellitus (N = 138)	93 (67.4%)	0.014	73 (78.5%)	0.003	20 (44.4%)	0.445	0.005
Chronic respiratory (N = 134)	91 (67.9%)	0.011	79 (77.5%)	0.004	12 (37.5%)	0.826	0.021
Chronic liver disease (N = 121)	76 (62.8%)	0.231	72 (67.9%)	0.444	4 (26.7%)	0.305	0.820
Chronic nephropathy (N = 41)	29 (70.7%)	0.086	26 (74.3%)	0.221	3 (50.0%)	0.588	0.241
Oncological (N = 183)	123 (67.2%)	0.005	104 (73.8%)	0.013	19 (45.2%)	0.397	0.012
Haemato-oncological (N = 19)	15 (78.9%)	0.059	13 (81.2%)	0.160	2 (66.7%)	0.329	0.150
Neurological (N = 237)	157 (66.2%)	0.003	146 (69.9%)	0.067	11 (39.3%)	0.999	0.106
Neurovascular (N = 53)	36 (67.9%)	0.124	31 (70.5%)	0.404	5 (55.6%)	0.311	0.300
Psychiatric (N = 290)	186 (64.1%)	0.011	169 (67.9%)	0.198	17 (41.5%)	0.761	0.221
Immuno-suppression (N = 53)	35 (66.0%)	0.210	31 (73.8%)	0.200	4 (36.4%)	0.839	0.373
Total diseases > 2 (N = 339)	245 (72.3%)	<0.0001	211 (77.9%)	<0.0001	34 (50.0)	0.041	<0.0001
Previous Herpes Zoster (N = 93)	54 (58.1%)	0.945	35 (60.3%)	0.482	19 (54.3%)	0.054	0.608

BMI = body mass index; * sufficient economic resources; ** physical activity present.

**Table 5:** Association between socio-demographic and clinical variables of the studied population (N = 1152) and occurrence of non-fatal acute vascular events starting from the 65th year onward (N = 42). The percentages apply to the column total. Statistical analysis was performed by means of the Chi-square test.

Socio-Demographic and Clinical Variables	Total	Event − N = 1110		Event + N = 42		*p*
	N (%)	N	%	N	%	
Male gender	520 (45.1%)	492	44.3	28	66.7	0.004
Age > 70 years	312 (27.1%)	292	26.3	20	47.6	0.002
Non-Caucasian ethnicity	9 (0.8%)	9	0.8	0	0.0	0.558
BMI > 30 kg/m^2^	184 (16.0%)	176	15.9	8	19.0	0.579
Smoking history	496 (43.1%)	472	42.5	24	57.1	0.060
Alcohol intake > 40 g/d	63 (5.5%)	60	5.4	3	7.1	0.627
Double dependency	27 (2.3%)	25	2.3	2	4.8	0.291
Not living alone	905 (78.6%)	874	78.7	31	73.8	0.445
Sufficient economic resources	1100 (95.5%)	1063	95.8	37	88.1	0.019
High educational level	574 (49.8%)	555	50.0	19	45.2	0.545
Physical activity present	542 (53.0%)	526	47.4	16	38.1	0.236
Total deficiencies ≤ 1	685 (40.5%)	663	59.7	22	52.4	0.341
Legal disability	64 (5.6%)	55	5.0	9	21.4	<0.0001
Institutionalized	5 (0.4%)	5	0.5	0	0.0	0.663
Arterial hypertension	614 (53.3%)	583	52.5	31	73.8	0.006
Cardiovascular disease	304 (26.4%)	275	24.8	29	69.0	<0.0001
Diabetes mellitus	138 (12.0%)	132	11.9	6	14.3	0.639
Chronic respiratory	134 (11.6%)	126	11.4	8	19.0	0.127
Chronic liver disease	121 (10.5%)	118	10.6	3	7.1	0.469
Chronic nephropathy	41 (3.6%)	38	3.4	3	7.1	0.202
Oncological	183 (15.9%)	173	15.6	10	23.8	0.152
Haemato-oncological	19 (1.6%)	18	1.6	1	2.4	0.704
Neurological	237 (20.6%)	223	20.1	14	33.3	0.037
Neurovascular	53 (4.6%)	39	3.5	14	33.3	<0.0001
Psychiatric	290 (25.2%)	280	25.2	10	23.8	0.836
Immuno-suppression	53 (4.6%)	51	4.6	2	4.8	0.959
Total diseases > 2	339 (29.4%)	311	28.0	28	66.7	<0.0001

BMI = body mass index.

**Table 6:** Results of Cox proportional hazard model to assess variables able to independently predict occurrence of acute vascular events. All the variables present in Table 5 plus past occurrence of Herpes Zoster and Herpes Zoster vaccination were included in the model.

Socio-Demographic and Clinical Variables	Coefficient	S.E.	Coefficient/SE	O.R.	95% C.I.	*p*
Cardiovascular disease	1.5666	0.3449	4.5424	4.7902	2.436–9.418	<0.001
Chronic nephropathy	1.2249	0.6141	1.9948	3.4039	1.021–11.342	0.087
Neurological	0.7924	0.3334	2.3765	2.2086	1.149–4.245	0.020
Neurovascular	1.8725	0.3446	5.4334	6.5043	3.310–12.780	<0.001
Herpes Zoster vaccination	−1.4705	0.4440	−3.3123	0.2298	0.096–0.549	<0.001

S.E. = standard error; O.R. = odds ratio; C.I. = confidence interval.

## Data Availability

The datasets generated during the current study are available from the corresponding author on reasonable request. Access to the data will be provided if considered justified by the journal’s editors or reviewers.

## Abbreviations

The following abbreviations are used in this manuscript:

HZ	Herpes Zoster
GP	General practitioner
ASAP	Experimental primary care outpatients’ clinics
